# The αMSH-Dependent PI3K Pathway Supports Energy Metabolism, via Glucose Uptake, in Melanoma Cells

**DOI:** 10.3390/cells12071099

**Published:** 2023-04-06

**Authors:** Giorgia Cardinali, Daniela Kovacs, Sarah Mosca, Barbara Bellei, Enrica Flori, Aldo Morrone, Anna Maria Mileo, Vittoria Maresca

**Affiliations:** 1 Laboratory of Cutaneous Physiopathology, San Gallicano Dermatological Institute, IRCCS, 00144 Rome, Italy; giorgia.cardinali@ifo.it (G.C.); daniela.kovacs@ifo.it (D.K.); sarah.mosca@ifo.it (S.M.); barbara.bellei@ifo.it (B.B.); enrica.flori@ifo.it (E.F.); aldo.morrone@ifo.it (A.M.); 2 Tumor Immunology and Immunotherapy Unit, Department of Research Advanced Diagnostic and Technological Innovation, Regina Elena National Cancer Institute, IRCCS, 00144 Rome, Italy

**Keywords:** melanoma, PI3K pathway, αMSH, glucose uptake, energy metabolism

## Abstract

Stimulation of melanocytes and murine melanoma cells with αMSH plus the PI3K inhibitor LY294002 resulted in ROS increase, oxidative DNA damage, and pigment retention. We performed cellular and molecular biology assays (Western blot, FACS, immunofluorescence analysis, scratch assay) on murine and human melanoma cells. Treatment with αMSH plus LY294002 altered cortical actin architecture. Given that cytoskeleton integrity requires energy, we next evaluated ATP levels and we observed a drop in ATP after exposure to αMSH plus LY294002. To evaluate if the αMSH-activated PI3K pathway could modulate energy metabolism, we focused on glucose uptake by analyzing the expression of the Glut-1 glucose translocator. Compared with cells treated with αMSH alone, those exposed to combined treatment showed a reduction of Glut-1 on the plasma membrane. This metabolic alteration was associated with changes in mitochondrial mass. A significant decrease of the cell migratory potential was also observed. We demonstrated that the αMSH-dependent PI3K pathway acts as a regulator of energy metabolism via glucose uptake, influencing the actin cytoskeleton, which is involved in melanosome release and cell motility. Hence, these results could constitute the basis for innovative therapeutical strategies.

## 1. Introduction

The melanocortin 1 receptor (MC1R), a G-protein-coupled receptor (GPCR), primarily located at the surface of melanocytes and melanoma cells, plays a crucial role in regulating the wide range of pigmentation degrees in mammals [1,2,3,4,5]. Several hypo-functional isoforms of this receptor, which increase skin photo-susceptibility and melanoma risk, have been described [1,6].

The interaction of MC1R with the α-melanocyte-stimulating hormone (αMSH) stimulates cAMP synthesis via Gs-protein, which in turn mediates the phosphorylation of the cAMP-responsive element-binding protein (CREB) transcription factor. CREB participates in the activation of the microphthalmia transcription factor (MITF), a key regulator of the expression of enzymes involved in melanogenesis [7,8,9]. We discovered the αMSH/peroxisome proliferator-activated receptor-γ (PPARγ) connection in melanoma cell lines and primary cultures of human melanocytes [10,11]. PPARγ belongs to the PPAR family. PPARs act as nuclear receptors. After activation, they translocate into the nucleus, form heterodimers with retinoic X receptors, and promote transcription of downstream target genes involved in lipid metabolism, maintenance of metabolic homeostasis, as well as in anti-inflammatory and anti-proliferative effects in a variety of human tumors [12,13].

More recently, we discovered a relationship between αMSH exposure and PI3K activation [14]. So far, few studies have addressed the role of the PI3K pathway in the biology of activated MC1R [14,15,16,17,18]. We described the involvement of PI3K in the αMSH-dependent melanogenesis and melanin distribution [14]. In fact, after PI3K inhibition, αMSH exposure generated large clusters of melanin in B16-F10 cells. In normal human melanocytes and in the ex vivo skin samples, the release of the pigment outside the cell was hindered. Moreover, the same treatment caused both an increase in reactive oxygen species (ROS) and DNA damage.

The PI3K pathway exerts important biological effects, such as improving cell viability and inhibiting senescence, aging, and death. In cancer cells, PI3K is often mutated and hyperactive and promotes anabolic metabolism, proliferation, survival, epithelial-to-mesenchymal transition (EMT), and chromosome instability [19,20]. These pleiotropic and highly energetic activities necessitate the intake and consumption of metabolic fuels, such as glucose molecules [21,22]. In cancer cells, including melanoma, glucose is mainly metabolized by aerobic glycolysis (the Warburg effect) and efficiently produces ATP and lactate as by-products. The Warburg effect is associated with increased rates of cell proliferation [23,24,25].

Starting from this background, in this study, we characterized the role of PI3K in glucose metabolism rearrangement induced by αMSH-mediated MC1R stimulation in melanoma cell lines. Herein, we provide evidence that the PI3K pathway is closely related to the membrane translocation of glucose transporter Glut-1, thus affecting the glucose consumption for mitochondrial ATP production. From our data, it can be argued that the αMSH/MC1R axis, via PI3K pathway activation, may support the melanocyte metabolic needs for highly energetic activities, such as melanosome trafficking and cell motility.

## 2. Materials and Methods

### 2.1. Cell Cultures and Treatments

The B16-F10 cell line is a classical melanoma cell line widely employed in pigmentation studies because it expresses a wild-type MC1R, with intact transduction machinery, which is activated in response to receptor stimulation [26]. B16-F10 mouse melanoma cells were cultured in Dulbecco’s modified Eagle’s medium (DMEM) supplemented with heat-inactivated 7% fetal bovine serum (FBS) and antibiotics (all products purchased by EuroClone, Milan, Italy). The human Mel16 melanoma cell line was isolated in our laboratory as previously reported [27], exclusively from excess parts of the biopsy collected for histological examination, without compromising the standard diagnostic procedure. Mel16 was isolated from a primary melanoma excised in the abdominal area of a 46-year-old female (pT4bN3M1, stage IVB). Molecular characterization by direct sequence analysis demonstrated that BRAF and NRAS genes are wild-type in this cell line. Mel16 was grown in OptiMEM (Invitrogen Life Technologies Italia, Monza, Italy) medium containing 10% FBS and antibiotics. Cell lines were seeded in a complete medium. After 24 h, they were kept overnight in a serum-free medium and then stimulated with chemicals in a fresh complete medium, for different lengths of time (see Results section). The αMSH was employed at a dose of 10^−7^ M. The LY294002, a PI3K inhibitor [28], was used at a concentration of 10 µM. In the αMSH plus LY294002 combined treatment, cells were pre-treated with PI3K inhibitors for 1 h. All compounds were purchased by Sigma-Aldrich Srl, Milan, Italy.

### 2.2. Cell Viability

Cells treated with different chemicals (see above) were detached by trypsinization and counted (in the presence of trypan blue to evaluate cell viability) using a phase-contrast microscope Axiovert 40C (Zeiss, Milan, Italy). Results are the average of three experiments performed in triplicate.

### 2.3. Cellular ATP Content

Cellular ATP was measured using the CellTiter-Glo Luminescent Assay (Promega, Madison, WI, USA) according to the manufacturer’s instructions. Cells were seeded at the concentration of 20,000–30,000 cells/mL into 96-well plates and treated based on the previously described protocol. After treatment, CellTiter-Glo reagent (20 μL) was added directly into each well and incubated for 10 min prior to reading the plate using a GLOMAX luminometer (Promega). The ATP content was calculated by comparing the luminescence levels of cells with those of control samples. The experiments were repeated 2 to 5 times. In all experiments, each sample was evaluated in 10–15-plicate.

### 2.4. Western Blot Analysis

B16-F10 and Mel16 melanoma cells were lysed in RIPA lysis buffer (Boster Biological Technology Co., Pleasanton, CA, USA) supplemented with a protease/phosphatase inhibitor cocktail (Boster Biological Technology Co.), then sonicated. Total cell lysates were clarified by centrifugation at 12,000 rpm for 10 min at 4 °C and then stored at −80 °C until analysis. Following spectrophotometric protein measurement, equal amounts of protein were resolved on acrylamide SDS-PAGE, and transferred onto nitrocellulose membrane (Amersham Biosciences, Milan, Italy). Protein transfer efficiency was checked with Ponceau S staining (Sigma-Aldrich, St Louis, MO, USA). Membranes were first washed with water, blocked with EveryBlot Blocking Buffer (Bio-Rad Laboratories Srl, Milan, Italy) for 10 min at room temperature, and then treated overnight at 4 °C with anti-glucose transporter Glut-1 rabbit monoclonal (ab115730, Abcam, Cambridge Science Park, Cambridge, UK), anti-phospho-Akt (Ser473, #4060, Cell Signaling Technology, Inc., Danvers, MA, USA), anti-Akt (#2920, Cell Signaling Technology, Inc.), and anti-mitofusin-2 (D1E9) rabbit monoclonal (#11925, Cell Signaling Technology, Inc.). Secondary anti-mouse IgG HRP-conjugated antibody (Cell Signaling Technology, Inc.) and anti-rabbit IgG HRP-conjugated antibody (Cell Signaling Technology, Inc.) were used. Antibody complexes were visualized using ECL (Santa Cruz Biotechnology Inc., Santa Cruz, CA, USA). A subsequent hybridization with monoclonal anti-β actin antibody (#A5441, Sigma-Aldrich, Milan, Italy) and GAPDH (#G9545, Sigma-Aldrich) were used as loading controls. Protein levels were quantified by measuring the optical densities of specific bands using the UVI-TEC Imaging System (Cambridge, UK). Densitometric results are expressed as fold change relative to the untreated sample value, which was set as 1.

### 2.5. Immunofluorescence Analysis

B16-F10 and Mel16 melanoma cells were fixed either with 4% paraformaldehyde followed by 0.1% Triton-X-100 to allow permeabilization or with cold methanol at −20 °C. Cells were then incubated with the primary antibody anti-glucose transporter Glut-1 rabbit monoclonal (1:100) (ab115730, Abcam, Cambridge Science Park, Cambridge, UK). The primary antibody was visualized using a goat anti-rabbit Alexa Fluor 546 conjugate (1:800) (ThermoFisher Scientific, S.r.l. Rodano, Milan, Italy). For immunofluorescence staining of the F-actin, the cells were incubated with TRITC-phalloidin (1:400) (P1951, Sigma-Aldrich). Coverslips were then mounted using ProLong Gold antifade reagent with DAPI (InVitrogen, Life Technologies, Monza, Italy). Samples were examined with an Apotome system (Zeiss, Oberkochen, Germany) connected to an Axio Observer inverted fluorescence microscope (Zeiss). Quantitative analysis of fluorescence signal was performed using the Zen 2.6 (blue edition) software (Zeiss). The results are expressed as mean fluorescence intensity/cell ± SD relative to the untreated sample value, which was set as 1.

### 2.6. Analysis of Mitochondrial Mass

MitoTracker Green FM (ThermoFisher Scientific) has been used to measure mitochondrial mass by flow cytometry. Cells were incubated with pre-warmed MitoTracker staining solution (diluted in phenol red-free medium to a final concentration of 10 nM) for 30–60 min at 37 °C. All subsequent steps were performed in the dark. Unstained cells were used as a negative control. Cells were washed in PBS, harvested, and re-suspended in 500 μL of PBS. At least 50,000/sample cells were analyzed using a MACSQuant10 instrument. Data were interpreted with MACSQuantify software (Milthenyi Biotech, S.r.l., Bologna, Italy).

### 2.7. Scratch Assay

B16-F10 and Mel16 melanoma cells were seeded on 35 mm dishes and allowed to grow until confluence. The cell monolayer was then wounded using a pipette tip (1 mL) to create a standardized cell-free area. After repeated washes, the cultures were then treated with αMSH, LY294002, and αMSH plus LY294002. Samples were fixed immediately after the scratch (T0) and after 24, 48, and 72 h. Images were recorded using a CCD camera (Zeiss) and migration was evaluated by measuring the edge distance using the Zen 2.6 software (Zeiss). The results are expressed as a percentage of reduction with respect to T0. 

### 2.8. Statistical Analyses

Results are expressed as means ± SD. Comparison among groups was performed by either Student’s test or ANOVA followed by Tukey’s multiple comparison test using GraphPad Prism (GraphPad Prism 8.0.2 Software, Boston, MA, USA). Detailed analyses of variance are reported in the legends of figures. Statistical significance for all tests was accepted for *p* < 0.05.

## 3. Results

### 3.1. The αMSH-Mediated PI3K Pathway Exerts a Key Role in Maintaining the Cytoskeletal Integrity

Our previous study demonstrated that the combined treatment with αMSH plus the PI3K inhibitor LY294002 caused a pigment retention phenomenon in B16-F10 melanoma cells, normal human melanocytes, and ex vivo skin samples [14]. Here, we demonstrated the pigment retention also on the human melanoma cell line Mel16 and this effect was associated with a significant reduction in the proliferation rate (Supplementary Figure S1).

**Figure 1:**
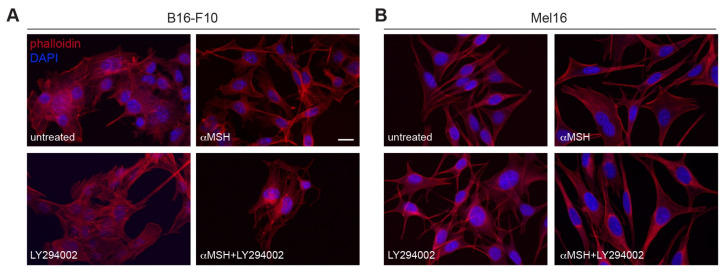
The αMSH-mediated PI3K pathway preserves cytoskeleton integrity. Immunofluorescence analysis with TRITC-phalloidin to reveal actin organization. B16-F10 (**A**) and Mel16 melanoma cells (**B**) were treated with αMSH, LY294002, and αMSH plus LY294002 for 72 h. Nuclei were counterstained with DAPI. Scale bar: 20 μm.

Actin and microtubule components of the cytoskeleton structure play a key role in the processes of dendrite extension and melanosome transport in melanocytes. In particular, actin-rich subcortical meshwork in the peripheral dendrite is involved in melanosome transfer to surrounding keratinocytes [29,30]. B16-F10 exposed to αMSH for 72 h displayed well-organized central stress fibers and peripheral/sub-cortical actin (Figure 1A). The organized bundles of actin filaments and cortical actin were severely affected in B16-F10 cells exposed to a combined treatment of αMSH plus LY294002 and F-actin staining appeared as brightly fluorescent clusters. Parallel analysis in the human melanoma cell line Mel16 (Figure 1B) showed alterations in actin structure, even if less pronounced in comparison with those observed in B16-F10. These results suggest that cytoskeleton modifications induced by the combined treatment are crucially involved in the observed melanosome retention. 

### 3.2. The αMSH-Dependent PI3K Pathway Exerts a Crucial Role in ATP Intracellular Production

**Figure 2:**
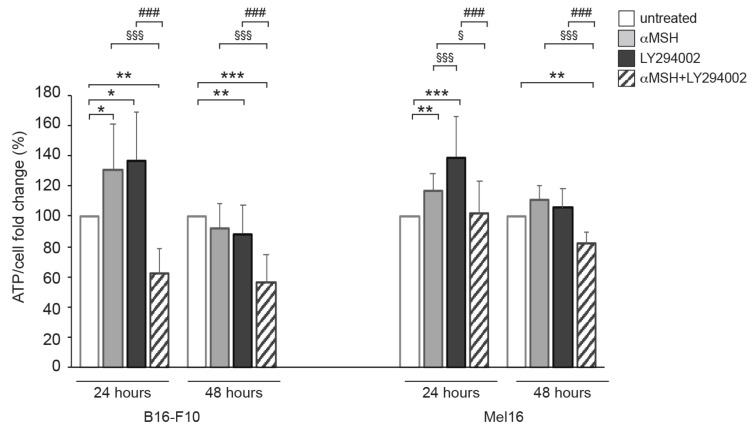
The αMSH-mediated PI3K pathway affects intracellular ATP content. B16-F10 and Mel 16 cell lines were treated with αMSH, LY294002, and αMSH plus LY294002 for 24 and 48 h and tested for intracellular ATP levels. ATP content is expressed as ATP/cell ratio and reported in the histogram. * *p* < 0.05, ** *p* < 0.01, *** *p* < 0.001 vs. untreated; ^§^ *p* < 0.05, ^§§§^ *p* < 0.001 vs. αMSH; ^###^ *p* < 0.001 vs. LY294002.

Melanosome traffic and transfer from melanocytes to keratinocytes are ATP-dependent processes. Given that PI3K is related to energy metabolism in several cell lines, including melanoma cells [21,22], an analysis of ATP levels was performed on cells exposed to αMSH for 24 h and 48 h, in the presence or absence of LY294002 (Figure 2). In murine melanoma B16-F10 and human melanoma Mel16 cell lines, the treatments with αMSH or LY294002 induced an increase in ATP levels after 24 h. By contrast, the combined treatment caused a significant drop in ATP levels in comparison with the samples treated with LY294002 or αMSH alone at both 24 and 48 h. These results showed that the αMSH-dependent PI3K pathway exerts a crucial role in ATP intracellular production.

### 3.3. The αMSH-Mediated PI3K Pathway Affects Membrane Recycling of the Glut-1 Transporter 

**Figure 3:**
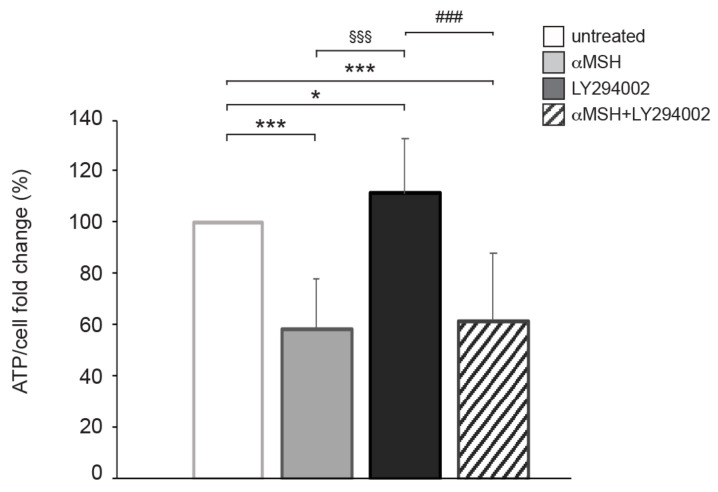
The αMSH-mediated PI3K pathway affects ATP content in cells cultured in a low-glucose medium. B16-F10 cells cultured in a low-glucose (1000 mg/L) culture medium were treated with αMSH, LY294002, and αMSH plus LY294002 for 24 h. * *p* < 0.05, *** *p* < 0.001 vs. untreated; ^§§§^ *p* < 0.001 vs. αMSH; ^###^ *p* < 0.001 vs. LY294002.

Given that the αMSH-dependent PI3K pathway played a crucial role in ATP intracellular production, we investigated whether this pathway may be involved in regulating glucose uptake. The intake of glucose occurs thanks to specific transporters (Gluts) expressed on the plasma membrane, which act as passive channels without requiring ATP [22]. We hypothesized that the αMSH-dependent PI3K pathway could be connected with the recycling of Gluts, thus allowing the entry of glucose and its conversion into ATP. To verify this hypothesis, we employed an indirect approach by analyzing ATP modulation on the B16-F10 cell line grown for 24 h in a culture medium containing a low glucose concentration (1000 mg/L) (Figure 3). In these conditions, after stimulation with αMSH alone, ATP levels significantly dropped and this effect was similar to that observed after combined treatment in cells grown under regular glucose (4500 mg/L) conditions (see above). These results demonstrate that in response to αMSH, the PI3K/AKT pathway is involved in the uptake of glucose in melanoma cells. 

**Figure 4:**
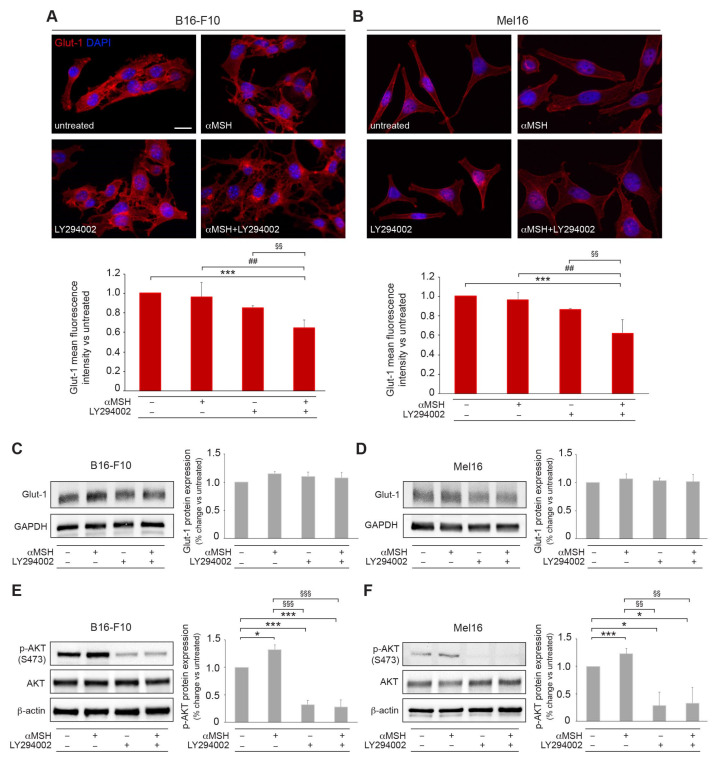
The αMSH-mediated PI3K pathway is involved in Glut-1 localization and recycling. Immunofluorescence and quantitative image analysis of Glut-1 localization in B16-F10 (**A**) and Mel16 melanoma cells (**B**) treated with αMSH, LY294002, and αMSH plus LY294002 for 6 h. Nuclei were counterstained with DAPI. Scale bar: 20 μm. Western blot and densitometric analysis of Glut-1 and phospho-Akt (S473) protein expression in B16-F10 (**C**,**E**) and Mel16 melanoma cells (**D**,**F**) treated with αMSH, LY294002, and αMSH plus LY294002 for 6 h. * *p* < 0.05, *** *p* < 0.001 vs. untreated; ^§§^ *p* <0.01, ^§§§^ *p* < 0.001 vs. αMSH; ^##^ *p* < 0.01 vs. LY294002.

Among the different glucose transporters, attention has been paid to Glut-1 which is involved in the metabolism of different tumors, including melanoma [31]. The localization of Glut-1 in response to αMSH, LY294002, or αMSH plus LY294002 was investigated in both B16-F10 and Mel16 cell lines by immunofluorescence analysis. In control and αMSH-treated cells, Glut-1 appeared clearly localized at the cell surface. The treatment with LY294002, and the combined treatment with αMSH, resulted in an evident reduction of the Glut-1 signal at the plasma membrane showing cytoplasmic localization, mainly in the perinuclear area. The measurement of the membrane signal intensity was assessed by image analysis and the results demonstrated a significant decrease in the fluorescence signal only following the combined treatment. (Figure 4A,B). However, in both melanoma cell lines, Western blot analysis did not show any significant difference in the overall Glut1 expression level, (Figure 4C,D). Given that Glut-1 activity is partly regulated by the activation of the PI3K/AKT pathway [21,32,33,34,35,36], we tested the pAKT (Ser 473) expression levels on B16-F10 and Mel16 after 6 h of stimulation with the different treatments (Figure 4E,F). As expected, both LY294002 and combined treatment significantly decreased pAKT expression levels, suggesting that the modulation of Glut-1 recycling at the plasma membrane is driven by PI3K signaling pathway.

**Figure 5:**
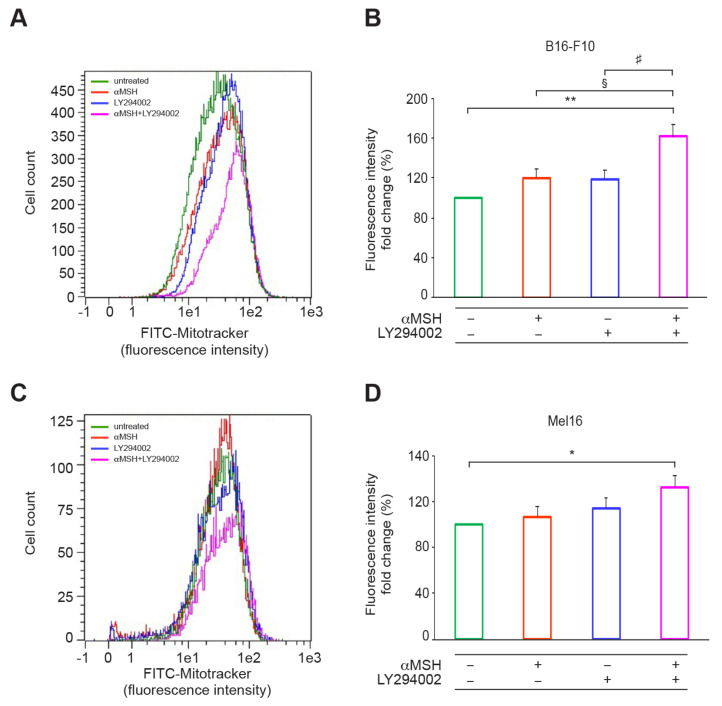
The αMSH-mediated PI3K pathway affects mitochondrial activity. Mitochondrial mass was evaluated by FACS analysis using MitoTracker Green FM staining. Histogram plots show the sample overlays of one representative experiment performed in B16-F10 (**A**) and Mel16 (**C**) cells at 24 and 48 h, respectively. (**B**,**D**) Histograms represent the mean ± SD of three independent experiments. * *p* < 0.05, ** *p* < 0.01 vs. untreated; ^§^ *p* < 0.05 vs. aMSH; ^#^ *p* < 0.05 vs. LY294002.

A state of glucose depletion causes a metabolic shift from glycolysis to oxidative phosphorylation, as a compensatory strategy for metabolic needs [24,37], as well as an increase in mitochondrial mass [25]. Given that the combined treatment mimicked a condition of glucose deprivation, we performed a mitochondrial mass analysis in both B16-F10 and Mel16. On B16-F10, the exposure to combined treatment of αMSH plus LY294002 caused a significant increase in this parameter in comparison with untreated and αMSH- or LY294002-treated samples. In Mel16 cells, the sample treated with αMSH plus LY294002 showed a significant increase in mitochondrial mass only versus the untreated sample (Figure 5).

In an attempt to strengthen the metabolic alterations following combined treatment (as evidenced by the ATP analysis) in Mel16, we evaluated the mitochondrial status by analyzing Mitofusin-2 expression (Supplementary Figure S2). The results we obtained evidenced a mitochondrial derangement, which does not allow the full recovery of energetic activity.

### 3.4. In Response to αMSH, the PI3K Pathway Affects Cell Motility

**Figure 6:**
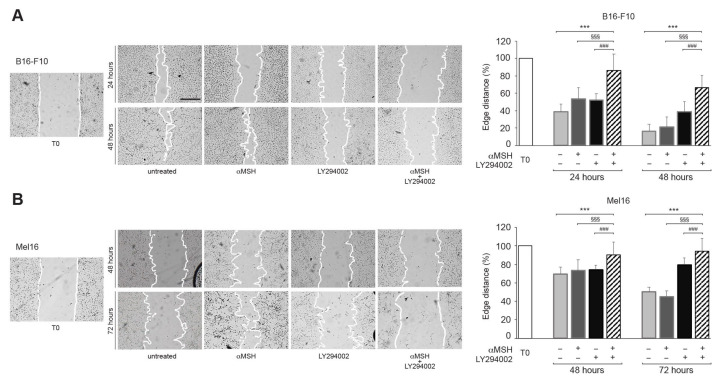
The αMSH-mediated PI3K pathway affects cell motility. Representative images of the scratch wound assay of B16-F10 (**A**) and Mel16 melanoma cells (**B**) treated with αMSH, LY294002, and αMSH plus LY294002 for 24, 48, and 72 h and quantitative analysis of the reduction of the edge distance in comparison to T0 (immediately after the scratch). Scale bar: 100 μm. *** *p* < 0.001 vs. untreated; ^§§§^ *p* < 0.001 vs. αMSH; ^###^ *p* < 0.001 vs. LY294002.

As cytoskeleton organization is known to affect cell motility and, consequently, tumor invasiveness and metastasis spread, we investigated the effects of the combined treatment with αMSH plus LY294002 on the motogenic activity of melanoma cells. A wound scratch assay was performed on B16-F10 cells and cellular migration was evaluated toward the scratched area after 24 and 48 h (Figure 6A). Quantitative analysis of the distance of the leading edges of the scratched area revealed a larger covered surface in all conditions compared to T0 at both time points evaluated. However, the area covered by the cells was significantly reduced in the cultures treated with αMSH plus LY294002 in comparison with the other experimental conditions. Similarly, the scratch assay performed on the Mel16 (Figure 6B) showed a reduction in the distance of the leading edge after all time points evaluated (48 and 72 h) in comparison to T0. Again, the measurement of the covered scratched area demonstrated a significantly smaller reduction in Mel16 co-treated with αMSH and LY29400 than in the other conditions. These results indicate that the combined treatment strongly inhibits the migratory potential of melanoma cells.

## 4. Discussion

We recently described how stimulation of B16-F10 melanoma cell line, human melanocytes, and human ex vivo skin with αMSH, in the presence of the PI3K inhibitor LY294002, resulted in reactive oxygen species increase, oxidative DNA damage, and evident accumulation of intracellular pigment, which was associated with a significant up-regulation of MITF expression, the main regulator of pigmentation [14], able to reduce migrative and invasive properties of melanoma cells [38]. Here, phase contrast analysis confirmed the pigment retention after αMSH plus LY294002 combined treatment, also in the human melanoma cell line Mel16. This effect was associated with the reduction of proliferation rate, not dissimilar to that observed in response to treatment with LY294002 alone. This latter aspect was not surprising because cancer cells are geared toward biomass production and proliferation, effects in which PI3K exerts a crucial role [39]. 

In an attempt to explain the phenomenon of melanin retention, we focused on the actin cytoskeleton organization, which is critical for melanosome traffic and transfer [29,30]. Melanoma cell lines treated with αMSH showed the actin cytoskeleton well-assembled in linear filaments and cortical rings. By contrast, cell lines exposed to combined treatment, showed an alteration in the architecture of cortical actin strictly involved in the release of melanosomes. This effect was dramatic in B16-F10 and less evident, albeit present, in Mel16. Nevertheless, in both melanoma cell lines, this alteration could account for the pigment retention phenomenon. 

The maintenance of cytoskeleton integrity greatly affects the energy balance in terms of ATP. In fact, it has to be continuously refueled and about 50% of cellular ATP is constantly employed for this purpose [40]. Other cellular activities, such as the synthesis of macromolecules and the maintenance of proliferation rate, can affect the energy balance [39]. Given that the duplication time of the Mel16 cell line is significantly higher than that of B16-F10 (30 vs. 18 h), this characteristic could justify a lower consumption of ATP and a less evident de-structuring effect of the actin cytoskeleton in comparison with the B16-F10 cell line. 

Cancer cells are committed to a strong anabolism. Glucose is their main carbon source. Its catabolism not only fuels ATP generation but also generates carbon intermediates to support the biosynthesis of macromolecules (i.e., lipids, proteins) [39]. The PI3K, highly active in tumors, is a cornerstone in glucose recruitment [41,42]. In association with activated tyrosine kinase receptors, PI3K promotes the expression of Glut-1 and its translocation from the intracellular membrane to the cell surface to facilitate glucose uptake [35,43]. Moreover, we previously demonstrated the activation of PI3K following prolonged stimulation of melanoma cells with αMSH [14]. 

Given that PI3K is related to energy metabolism in several cancer cells, including melanomas [21,22], an analysis of ATP was performed on cells exposed to αMSH in the presence or absence of LY294002. In response to αMSH exposure, the increase in ATP levels suggests that the hormone may favor glucose uptake for energy purposes. After αMSH plus LY294002 combined treatment, the drastic reduction of ATP levels underlines the fact that the αMSH-dependent PI3K activation exerted a key role in promoting glucose uptake. Furthermore, in both melanoma cell lines, the significant increase in ATP levels, observed after stimulation with LY294002 for 24 h, which returned to basal levels after 48 h, suggests that non-glucosidic compounds may be metabolized to generate energy and that these strategies may be only temporarily employed. Both αMSH and LY294002 induced an increase in ATP levels after 24 h, while the combined treatment instead causes its drastic reduction (earlier in B16-F10 and more delayed in Mel16). This aspect was somewhat puzzling. On the basis of the above, it is possible to hypothesize that the αMSH stimulus places the cells in a state of supplementary energy demand (necessary to perform melanogenesis, vesicle synthesis, and cytoskeleton modifications). When glucose uptake is reduced, due to the inhibition of the PI3K pathway, the additional energy requirement may not be satisfied solely by the catabolism of non-glucosidic compounds.

After ATP assessments, which indirectly suggest a link between PI3K activity and glucose uptake, some experiments were performed on B16-F10 grown in low glucose concentrations. In these culture conditions, ATP levels significantly dropped even after stimulation with αMSH alone (an effect that did not occur when culturing cells with standard glucose concentrations). It was possible to hypothesize that in these conditions, the cells, after hormonal exposure, could not recruit glucose from the external environment and fell into severe ATP deficiency. These results suggest that the αMSH exposure favored the entry of glucose to support the energy needs associated with the maturation processes of melanogenically active cells.

The melanocortin system is a well-established regulator of insulin secretion and glucose utilization [44,45,46]. Recently, it has been shown that αMSH promotes glucose uptake in the skeletal muscle acting on MC5R, a member of the same family of MC1R [47]. Moreover, the involvement of PI3K in regulating glucose uptake has been described in association with the presence of functional glucose transporters, such as Glut-1 and Glut-4 on the plasma membrane [32,33,34,35,48,49].

Glut-1 is characterized by the highest affinity for glucose and is responsible for its basal uptake in all tissues. Glut-1 is frequently found over-expressed in tumor cell membranes, including melanomas [31]. How melanoma cells manage to survive and proliferate by consuming glucose has been partially addressed in the literature, but some rather interesting results have been presented [50]. Different studies have demonstrated an Important connection between PI3K activity and membrane translocation of Gluts [21,32,33,34,35,36].

We focused on Glut-1 for its key role in tumors and tissues that are highly dependent on glucose energy [48]. In several cancers, including melanoma, its expression correlates with tumor progression and clinical outcome [31,48]. The expression of Glut-1 at the plasma membrane is the result of transporter trafficking. Glut-1 has two principal fates: either it is internalized inside vesicles to be recycled, therefore returning to the plasma membrane, or it is sorted to the lysosome for degradation [51]. In both melanoma cell lines, the combined treatment αMSH/LY294002 interfered with the Glut-1 localization without modifying Glut-1 protein expression levels, as elsewhere described [49]. Therefore, we can hypothesize that the ATP drop could be due to a reduced glucose uptake caused by the delocalization of Glut-1 from the plasma membrane to the intracellular pool.

Given that Gluts activity is regulated by PI3K/AKT pathway [21,32,33,34,35,36], pAKT (Ser 473) expression levels were monitored on B16-F10 and Mel16 cell lines after exposure to different treatments. Both LY294002 and combined treatments significantly decreased pAKT (Ser 473) expression levels, leading to the interruption of some steps responsible for the translocation of Glut-1 to the plasma membrane. 

Cell lines subjected to combined treatment were, therefore, in a condition of metabolic distress, with a reduced ability to uptake glucose and a deficiency of ATP. In this condition, the metabolism shifts from glycolysis to oxidative phosphorylation, as a compensative strategy [24,37]. Mitochondrial activity generates oxidative stress because mitochondrial complexes develop free radicals as a by-product of their activity [37]. These data were in agreement with our previous results showing a significant ROS increase in B16-F10 exposed to the same treatment [14]. Moreover, this metabolic condition justified the increase in mitochondrial mass (although ineffective) that we observed in both cell lines in response to αMSH plus LY294002. The different entity of mitochondrial mass increase (lower in Mel16) might be related to the modulation of Mitofusin-2 expression by LY294002, which indicates some different dynamic process involving mitochondrial shape remodeling. In fact, mitochondria are highly dynamic organelles, undergoing coordinated cycles of fission and fusion, referred as ‘mitochondrial dynamics’, in order to maintain their shape, distribution and size [52]. Their rapid morphological adaptations are crucial for many cellular processes, such as cell cycles, immunity, apoptosis, and mitochondrial quality control. Mitochondrial fission allows the division of one mitochondrion in two daughter mitochondria. Mitochondrial fusion is the confluence of two or more mitochondria. In this process, mitofusins 1 and 2 are involved. In this plasticity, the cytoskeleton also plays a relevant role in maintaining the mitochondrial network, which is tuned to cellular functions [53]. 

The actin cytoskeleton assembly is crucial also for migration and invasion and it is an energy-intensive process [54]. After combined treatment, a reduced cell migratory potential was observed (as assessed by the larger covered area measured by the wound scratch assay). We hypothesize that this effect may derive from the energy deficit due to the ATP drop. Given that tumor aggressiveness is driven by both cell growth and invasiveness, these data suggest an inhibiting effect of the combined treatment on the melanoma cell migratory properties involved in malignant spreading. 

## 5. Conclusions

**Figure 7:**
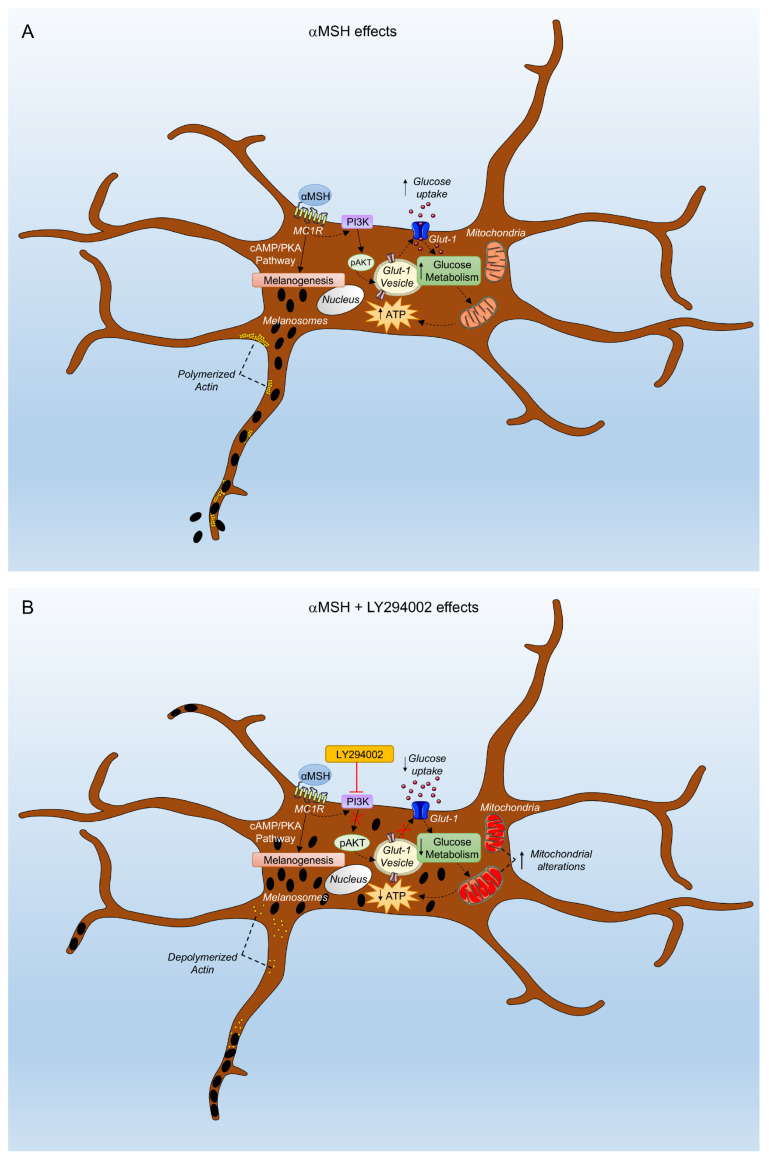
The αMSH-dependent PI3K pathway supports energy metabolism via glucose uptake in melanoma cells. (**A**) αMSH-dependent activation of PI3K signaling promotes glucose uptake by maintaining the Glut-1 transporter on the plasma membrane. (**B**) αMSH plus LY294002 treatment affects glucose uptake, determining mitochondrial derangement, reduced ATP production, and cytoskeleton modifications. Up and down arrows indicate an increase or decrease of the represented events, respectively.

In summary, we previously showed that in response to αMSH, the PI3K pathway exerted a role in promoting the extracellular release of pigment and in preserving redox equilibrium and genome integrity [14] in B16-F10, normal human melanocytes, and human skin ex vivo. Here, on melanoma cell lines, we additionally demonstrated that in response to αMSH, the PI3K pathway was involved in the recycling of Glut-1 on the plasma membrane, affecting glucose uptake and ATP production, thus acting as a regulator of energy metabolism (Figure 7). These effects, in turn, affected melanosomes release and cell motility. Given that melanoma has been emerging as a metabolic disorder [51], the identification of major metabolic alterations, as well as lipid metabolic networks that regulate melanoma growth and survival, could constitute a basis for the development of innovative therapeutic strategies.

## Data Availability

The datasets used or analyzed during the current study are available from the corresponding author upon reasonable request.

## Supplementary Materials

The following supporting information can be downloaded at: https://www.mdpi.com/article/10.3390/cells12071099/s1, Figure S1: The αMSH-mediated PI3K/AKT pathway influences dendricity, pigmentation, and cell proliferation of Mel16 melanoma cells.; Figure S2: Western blot and densitometric analysis of Mitofusin-2 expression in Mel16 cells treated with αMSH, LY294002 and αMSH plus LY294002 for 24 h.
