# Supplementary material for: The αMSH-Dependent PI3K Pathway Supports Energy Metabolism, via Glucose Uptake, in Melanoma Cells
Source: Cells. 2023 Apr 6;12(7):1099. doi: 10.3390/cells12071099 (PMC10093374; doi:10.3390/cells12071099)
Supplement: Supplementary file 1 [file cells-12-01099-s001.zip › cells-2225191-supplementary.pdf]

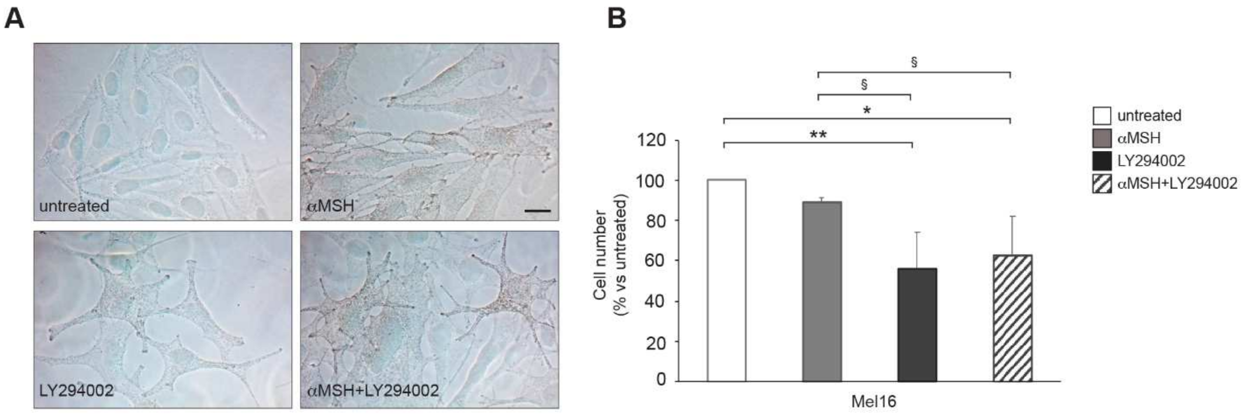

Supplementary Figure S1. The αMSH-mediated PI3K/AKT pathway influences dendricity, pigmentation and cell proliferation of Mel16 melanoma cells. Phase contrast analysis (A) and cell count (B) of Mel16 cells treated with αMSH in the presence or absence of LY294002 for 48 hours. Scale bar: 20 μm. *$p<0.05$, **$p<0.01$ vs untreated; $^{\S}p<0.05$ vs αMSH.

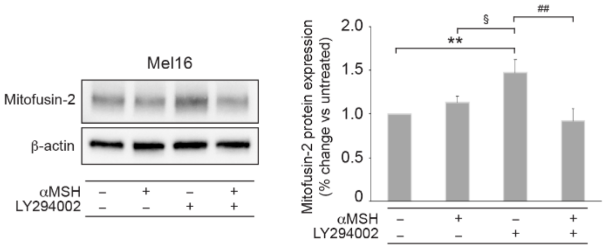

Supplementary Figure S2. Western blot and densitometric analysis of Mitofusin-2 expression in Mel16 cells treated with αMSH, LY294002 and αMSH plus LY294002 for 24 hours. **$p<0.01$ vs untreated; $^{\S}p<0.05$ vs αMSH; $^{\#\#}p<0.05$ vs LY294002.
